# Metabolic profiling of red-fleshed apple and functional characterization of *MdERF072* in anthocyanin biosynthesis

**DOI:** 10.1186/s12870-025-07987-5

**Published:** 2025-12-23

**Authors:** Yi Ren, Yi Guan, Yan Ma, Zian Fang, Yumei Ding, Pingxing Ao

**Affiliations:** 1 College of Landscape and Horticulture, Yunnan Agricultural University, Kunming, Yunnan 650201 China; 2 Yuanjiang County Planting Industry Development Service Center, Yuxi, Yunnan 653300 China

**Keywords:** Red-fleshed apple, Flavonoids, Proanthocyanidins, Polymethoxyflavones, AP2/ERF

## Abstract

**Background:**

Red-fleshed apples, with their flavonoid-rich edible flesh, offer superior nutritional advantages over traditional varieties, where these antioxidants are concentrated primarily in the less-consumed peel. The distinct metabolic profile of red-fleshed apples, particularly their compositional uniqueness and deposition characteristics, therefore requires systematic deconstruction through comparative metabolomics.

**Results:**

In this work, comparative metabolomics of three apple cultivars with distinct flesh coloration revealed a substantial upregulation of specialized flavonoids in the red-fleshed variety. Notably, we identified an accumulation of polymethoxyflavones (PMFs) with antitumor activity and found cyanidin-3-*O*-glucoside to be the predominant anthocyanin responsible for red pigmentation in red-fleshed apple. Conversely, the accumulation of flavan-3-ols and proanthocyanidins (PAs) was decreased in red-fleshed apple. Volatile metabolome analysis interestingly demonstrated that the majority of volatile organic compounds (VOCs), such as esters, terpenoids, and alcohols, were significantly more abundant in the red-fleshed apple. Transcriptomic profiling revealed the upregulation of key flavonoid pathway genes, including early synthase genes (*PAL*, *C4H*, *4CL*, *CHS*) and anthocyanin-specific genes (*UFGT*, *GST*), in red-fleshed apple, consistent with the observed accumulation of PMFs and anthocyanins in the flesh. Conversely, the downregulation of *LAR* and *ANR* accounts for the reduced biosynthesis of flavan-3-*ols* and PAs in the red-fleshed apple. Systematic TF screening, expression analyses and transient verification assays revealed that *MdERF072* acts as an important transcriptional activator of anthocyanin biosynthesis and potentially upregulates the expression of structural genes in the anthocyanin pathway.

**Conclusions:**

Overall, this work reveals the metabolic and transcriptional basis of flavonoid enrichment in red-fleshed apple and provides a foundation for breeding nutritionally improved cultivars.

**Supplementary Information:**

The online version contains supplementary material available at 10.1186/s12870-025-07987-5.

## Background

Anthocyanins, a class of flavonoid-derived secondary metabolites, contribute to plant coloration and serve as a major dietary source of antioxidants in fruits and vegetables [1]. They are also thought to play crucial roles in enhancing resistance to various abiotic and biotic stresses, such as drought, cold, and pathogen attack, by acting as scavengers of reactive oxygen species in plants [2, 3]. Anthocyanins accumulate in vacuolar compartments and generate coloration in horticultural crops, which spans from pink to purple, depending on the structural type and cellular pH conditions of various plant organs, including leaves, flowers, and fruits [4, 5]. In addition to the colorful appearance of fruit peels, growing attention is being given to pigments in fruit flesh owing to their role in enhancing the nutritional quality and health benefits of plant-derived foods. Therefore, numerous novel fruits with appealing flesh color and rich in flavonoids, such as blood orange [6, 7], purple kiwifruit (*Actinidia species*) [8], purple tomato [9], and red-fleshed peach [10], have garnered significant interest among both breeders and consumers.

The antioxidant capacity is a key property of apple fruits, which are rich in flavonoids and anthocyanins. Generally, the fruit flesh of most apple varieties is white or off-white in color, and the skin (or peel) is green or red. Over the past few decades, a number of red-fleshed *Malus* varieties originating from Central *Asia* have been found, and breeders around the word have bred red-fleshed apple varieties, such as ‘Red love’, ‘Pink Pearl’, ‘Weirouge’, ‘Rosette’, and ‘JPP35’ [11–14]. Red coloration in apple flesh and peel results from anthocyanin accumulation [15, 16]. Its formation occurs through a specialized branch of the flavonoid biosynthesis pathway, where the associated catalytic enzymes and their encoding genes have been extensively characterized in apple [1]. The ternary complex MYB/bHLH/WD40 (MBW), containing MYB transcription factors (TFs), basic helix-loop-helix (bHLH) TFs, and WD-repeat proteins, is considered the key regulatory machine controlling anthocyanin accumulation by regulating the expression of catalytic enzyme-encoding genes [17]. In the red-fleshed apple ‘Red Feild’ variety, which belongs to Niedzwetzky’s apple tree, a minisatellite-like structural variation that comprises five direct tandem repeats of a 23-bp sequence (R6:*MdMYB10*) in the upstream regulatory region of *MdMYB10* induces red foliage and red fruit flesh, whereas only one repeat is found in white-fleshed apple varieties (R1:*MdMYB10*) [11, 18]. The consistency between genotypes and phenotypes in F1 hybrid populations, which were constructed to breed and improve red-fleshed apple through crossing between *Malus sieversii* f. *niedzwetzkyana* (R6R1) and *M. domestica* (R1R1), has provided compelling evidence supporting this hypothesis [19]. However, phenotypic variation in flesh color was observed among R6R1 genotypes in F2 hybrid populations derived from crosses between R6R6-homozygous mutants and *M. domestica* (R1R1), indicating that the existence of additional regulatory factors controls apple flesh color variation [13]. Subsequent studies revealed that WRKY family TFs such as *MdWRKY11* and *MdWRKY10* are also involved in anthocyanin accumulation in apple flesh [13, 15]. Moreover, a 163-bp InDel with a W-box element is found in the promoter region of the alleles of *MdWRKY10,* which activates the expression of *MdMYB10* and enhances the transcriptional activation activity of the MBW complex [15]. This finding largely explains the degree of variation in flesh red pigmentation in apple.

In addition to the WRKY family, other TFs have been shown to participate in regulating anthocyanin accumulation in apple. The overexpression of *MdNAC1* in apple significantly promotes the accumulation of anthocyanins, where MdNAC1 activates the transcription of *MdMYB10* and *MdUFGT* by interacting with MdbZIP23 [20]. MdERF109 was found to promote coloration by directly binding to anthocyanin-related gene promoters in apple [5]. Another ERF family member, MdERF38, is involved in drought stress-induced anthocyanin accumulation by interacting with MdMYB1, a positive modulator of anthocyanin biosynthesis [21]. The overexpression of MdERF1B increased the transcription of flavonoid biosynthesis pathway-related genes such as *MdCHS*, *MdCHI*, *MdF3H*, *MdDFR*, *MdANS*, and *MdLAR* [22]. AP2/ERF transcription factors have been shown to regulate anthocyanin synthesis in other species. In lily, *LvERF113* promotes anthocyanin synthesis in lily tepals by inhibiting the negative regulatory role of *LvMYB1* [23]. In litchi, *LcERF1*/*22*/*25*/*37* govern anthocyanin biosynthesis in the pericarp by regulating the expression of anthocyanin biosynthesis genes [24]. Nevertheless, functional analyses have demonstrated that AP2/ERF family members exhibit differential regulatory effects on anthocyanin biosynthesis. The DREB member *LhERF061* suppresses anthocyanin biosynthesis by inhibiting the transcription of the positive regulators *LhMYBSPLATTER* and *LhDFR* in lily [25]. Therefore, we wondered whether additional AP2/ERF family members are involved in regulating flesh coloration in apple.

In addition to anthocyanin accumulation differences, the phytochemical profile results revealed that red-fleshed apples contained greater amounts of phenolic acids, dihydrochalcones, and organic acids; however, lower amounts of flavan-3-*ols*, which are precursors of proanthocyanidin biosynthesis, are found in red-flashed apples than in white-fleshed apples [14]. Proanthocyanidins (PAs), also known as condensed tannins, are polymerized from (*cis*-)*trans*-flavan-3-*ol* units that are produced via the flavonoid biosynthesis pathway and accumulate in vacuoles in plants. [26]. PAs are widely found in the leaves of tea, bark, and fruits of many horticultural plants, including apples, grapes, kiwifruit, persimmon, and blueberry [27–31], and contribute to the resistance of plants to biotic and abiotic stresses, leading to bitterness and astringency in food products [32]. In apple fruit, the flavonoid biosynthetic pathway has been well described, and *trans*- and *cis*-flavan-3-*ols* (catechin and epicatechin) have been shown to act as initiating units for the synthesis of PAs [33]. Several flavonoid biosynthesis pathway genes associated with anthocyanin synthesis are also required for the synthesis of PAs. However, the differences in the accumulation of PAs between red-fleshed apples and traditional white-fleshed apples have not been comprehensively investigated.

Another crucial quality attribute in apple is aroma, which is generally a complex mixture of volatile organic components (VOCs), exerting a substantial effect on their overall flavor profile and consumer preference [34]. Approximately 350 volatile compounds have been found in apples, and only a subset of 15–20 compounds, such as esters, aldehydes, alcohols, and terpenes, significantly contribute to the typical apple aroma [35]. Solid-phase microextraction combined with gas chromatography‒mass spectrometry (SPME‒GC‒MS) has been widely used to evaluate volatile profiles in apple. For example, the volatile profiles of the flesh of 85 apple cultivars have been evaluated, and 70 volatile compounds have been identified [36]. The aroma volatile compounds in the peels of 40 apple cultivars were also investigated, and 78 volatile compounds were identified [37]. Nevertheless, red-fleshed apples have garnered significant research interest owing to their elevated flavonoid levels [14]; consequently, their distinctive volatile profiles need to be further examined.

In this study, we comprehensively compared the distinct metabolic characteristics of the pulp tissues between red-fleshed apple cultivars and two traditional white-fleshed cultivars through an analysis of the whole profile of nonvolatile metabolites (flavonoids, phenolic acids, organic acids, tannins, etc.) and the whole profile of VOCs (terpenoids, esters, alcohols, etc.). Integrated with transcriptomic data and transient expression assays in apple, our results revealed that the AP2/ERF family member *MdERF072* positively regulates anthocyanin accumulation, likely through activating key genes involved in the flavonoid pathway. These findings collectively illustrate the unique metabolic signature of red-fleshed apples and identify a novel transcription factor that acts as transcriptional activators of anthocyanin biosynthesis.

## Materials and methods

### Plant materials

Three apple cultivars, namely, ‘Red Love 119–07’ (named ‘RF’ in the following text), ‘YanFu 8’ (named ‘MF’ in the following text), and ‘Venus Golden’ (named ‘YF’ in the following text), were used as materials to conduct widely targeted metabolomic and RNA-seq analyses. All apple varieties were planted at the Fruit Germplasm Resources Collection and Conservation Center [Yunnan, China (101°10′−103°40′E, 24°23′−26°33′N)]. All apple trees were managed under uniform horticultural methods until fruit reached maturity. At harvested, the fruits were sampled, photographed, and peeled. The flesh was frequently frozen in liquid nitrogen and stored at −80 °C for subsequent analysis.

### Metabolite extraction and LC‒MS/MS analysis

Metabolite extraction was performed following a previously described protocol with minor modifications [38]. Briefly, freeze-dried samples were finely ground, and 100 mg of the powder was subjected to overnight extraction using 1.0 mL of 70% aqueous methanol at 4 °C. Following centrifugation (10,000 × g, 10 min), the supernatants were purified via a CNWBOND Carbon-GCB SPE cartridge (250 mg, 3 mL; ANPEL, Shanghai, China) and filtered through a 0.22 μm membrane (SCAA-104, ANPEL, Shanghai, China) prior to LC‒MS analysis. LC‒MS analysis was conducted on an LC‒ESI‒MS‒MS system consisting of a Shim-pack UFLC SHIMADZU CBM30A HPLC (http://www.shimadzu.com.cn/) coupled with an Applied Biosystems 4500Q TRAP mass spectrometer (http://www.appliedbiosystems.com.cn/). The liquid chromatography conditions were adopted from an established method [39].

Mass spectrometric data were acquired via an API 4500 Q TRAP LC/MS/MS system (AB Sciex, USA) equipped with an ESI Turbo Ion Spray interface. The instrument was operated in both positive and negative ion modes under Analyst 1.6.3 software control (AB Sciex, Singapore). The ESI source operation parameters were as follows: ion source, turbo spray; source temperature, 550 °C; ion spray voltage (IS), 5500 V; and ion source gas I (GSI), gas II (GSII), and curtain gas (CUR) set to 55, 60, and 25 psi, respectively. The collision-activated dissociation (CAD) was set at “high”. For calibration, 10 and 100 μmol·L^−1^ polypropylene glycol solutions were used in triple quadrupole (QQQQ) and linear ion trap (LIT) modes, respectively. The quantitative analysis employed multiple reaction monitoring (MRM) with nitrogen at 5 psi, where the declustering potential (DP) and collision energy (CE) were individually optimized for each transition. Metabolite-specific MRM transitions were monitored on the basis of elution profiles, as previously described [38].

### Extraction of volatile organic components (VOCs) and GC‒MS/MS analysis

The samples were ground to powder in liquid nitrogen. 1 g of powder was transferred immediately to a 20 mL head-space vial (Agilent, Palo Alto, CA, USA) containing NaCl saturated solution to inhibit any enzyme reactions. The vials were sealed via crimp-top caps with TFE-silicone headspace septa (Agilent). At the time of SPME analysis, each vial was placed at 100 °C for 5 min, and then, a 120 µm divinylbenzene/carboxen/polydimethylsilioxan fiber (Agilent) was exposed to the headspace of the sample at 100 °C for 15 min to collect VOCs.

After sampling, desorption of the VOCs from the fiber coating was carried out in the injection port of the GC apparatus (Model 8890; Agilent) at 250 °C for 5 min in splitless mode. The identification and quantification of VOCs were carried out via an Agilent Model 8890 GC and a 5977B mass spectrometer (Agilent) equipped with a 30 m × 0.25 mm × 0.25 μm DB-5MS (5% phenyl-polymethylsiloxane) capillary column. Helium was used as the carrier gas at a linear velocity of 1.2 mL·min^−1^. The injector temperature was maintained at 250 °C, and the detector temperature was maintained at 280 °C. The oven temperature was programmed from 40 °C (3.5 min), increased at 10 °C·min^−1^ to 100 °C, increased at 7 °C·min^−1^ to 180 °C, increased at 25 °C·min^−1^ to 280 °C, and held for 5 min. Mass spectra were recorded in electron impact (EI) ionization mode at 70 eV. The quadrupole mass detector, ion source and transfer line temperatures were set at 150, 230 and 280 °C, respectively. The mass spectra were scanned in the range of m·z^−1^ 50–500 amu at 1 s intervals. The identification of volatile compounds was achieved by comparing the mass spectra with the data system library and linear retention index.

### MS data and statistical analyses

MS data were acquired and processed according to a previously described method [40]. Analyses of the primary and secondary MS data were conducted on the basis of the self-built database MWDB (MetWare Biotechnology Co., Ltd. Wuhan, China). Metabolite quantification was accomplished with data acquired in MRM mode by QQQ-MS [38]. The identification of differentially abundant metabolites was determined by an absolute log_2_FC (fold change) ≥ 1 and a VIP ≥ 1. VIP values were extracted from the OPLS-DA results generated via the R package MetaboAnalystR.

### Library preparation and RNA sequencing

The RNA sequencing libraries were prepared via the NEBNext® Ul-traTM RNA Library Prep Kit for Illumina® (USA, NEB) according to the kit instructions. Briefly, poly(A) mRNA was isolated via oligo(d_T_)-attached magnetic beads, followed by first-strand cDNA synthesis with random hexamer primers and M-MuLV Reverse Transcriptase (NEB, USA). Second-strand synthesis was performed via DNA polymerase I and RNase H, followed by end repair and adenylation of the 3’ ends. NEBNext adaptors were then ligated to the cDNA fragments. Size selection (250–300 bp) was performed via the AMPure XP system (Beckman Coulter, USA), followed by USER Enzyme (NEB) treatment at 37 °C for 15 min and 95 °C for 5 min. Then, a PCR assay was carried out with Phusion High-Fidelity DNA polymerase, Universal PCR primers, and Index (X) Primer. The final libraries were purified via AMPure XP beads and quality checked on an Agilent Bioanalyzer 2100 system before paired-end sequencing (125/150 bp) on an Illumina HiSeq2500 platform.

### Functional annotation and expression analysis

The raw sequencing data were processed to remove low-quality reads and adapters, generating high-quality clean reads. The HISAT v2.1.0 package was used to construct the index and map the clean reads to the NCBI GenBank. The feature Counts v1.6.2 package was used to count the gene alignment, and then the fragments per kilobase per million (FPKM) of each gene was calculated on the basis of the gene length. DESeq2 v1.22.1 was used to analyze the differential expression between the two groups, and the *p* value was corrected via the Benjamini & Hochberg method. Gene function was annotated according to the following databases: NCBI nonredundant protein sequences (Nrs), Clusters of Orthologous Groups of proteins (COG/KOGs), Swiss PROT protein sequence database (SwissProt), Kyoto Encyclopedia of Genes and Genomes (KEGG), homologous protein family (Pfam) and Gene Ontology (GO).

### Real-time quantitative PCR (RT‒qPCR) verification

The total RNA of apple plants was extracted via the FastPure Complex Tissue Total RNA Isolation Kit (Vazyme, China) and transcribed to cDNA via random primers (for all circRNA detection) via the HiScript III 1 st Strand cDNA Synthesis Kit (+ gDNA wiper) (Vazyme, China) in accordance with the manufacturer’s instructions. RT‒qPCR analysis was conducted to evaluate the expression levels of all genes associated with this work via SYBR qPCR Mix (Vazyme, China) with an ABI7500 Real-Time PCR Detection System (Thermo Fisher, USA). Gene expression levels were calculated via the 2^−∆∆CT^ method [41]. All primer pairs used in this work are listed in Table S7.

### Transient expression and gene function verification

The *MdERF072* gene was cloned and inserted into pHB with the CaMV 35S promoter and then transformed into *Agrobacterium tumefaciens* strain GV3101 via the freeze‒thaw method. *A. tumefaciens* was incubated at 28 °C for 2 d on LB media supplemented with 25 mg/L rifampicin and 50 mg/L kanamycin. A single clone was verified by PCR and incubated at 28 °C for 8–10 h with shaking. For transient expression in apple, a positive agrobacteria suspension was inoculated in 15 mL of LB liquid medium until the OD_600_ reached 0.6–0.8. After centrifugation, the pellet was resuspended, and the OD_600_ was adjusted to 0.2 with MAA buffer (10 mM MES, 10 mM MgCl_2_, 0.1 mM acetosyringone, pH 5.6) and injected into the ‘Fuji’ apple fruit through a syringe without a needle. *A. tumefaciens* carrying the pHB empty vector was used as a control. After infiltration, the fruits were placed in an incubator at 25 °C under 16 h light/8 h dark conditions. After 3 days, the fruit injection sites were sampled for transgene verification and RT‒qPCR analysis.

## Results

### Inter-cultivar metabolomic profiling of apple flesh

**Fig. 1:**
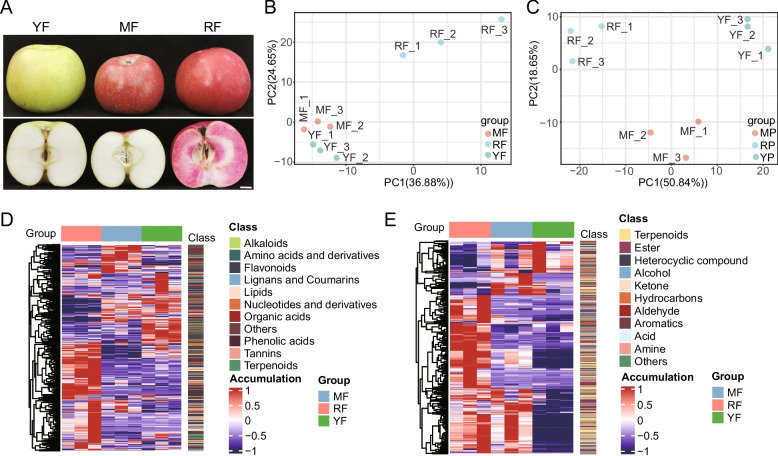
The outline of accumulated metabolites of three apple cultivars. **A** Morphological phenotypes of ‘YF’, ‘MF’, and ‘RF’ apple cultivars, bar = 1 cm. **B**, **C** Principal component analysis (PCA) results for three apple cultivars (three biological replicates) on the basis of LC‒MS/MS data (**B**) and GC‒MS/MS data (**C**). **D**, **E** Hierarchical clustering and classification of metabolites identified from three apple cultivars on the basis of LC‒MS/MS data (**D**) and GC‒MS/MS data (**E**)

In our previous work, we collected three apple cultivars with distinct peel and flesh coloration, namely, ‘MF’, ‘YF’, and ‘RF’. Among them, ‘RF’ has a red peel and flesh, ‘MF’ has a red peel and white flesh, and ‘YF’ has a green peel and off-white flesh (Fig. 1A). To comprehensively investigate the metabolic accumulation differences in the flesh of the three cultivars, metabolic profiling was performed to analyze both nonvolatile metabolites and volatile organic compounds. In the present work, a total of 705 nonvolatile metabolites were identified from three samples via a UPLC‒MS/MS system (Table S1). According to the metabolomic data, nonvolatile metabolites in apple flesh were divided into the following categories: 139 (19.72%) flavonoids, 128 (18.16%) phenolic acids, 103 (14.61%) lipids, 62 (8.79%) organic acids, 60 (8.51%) amino acids and derivatives, 39 (5.53%) nucleotides and derivatives, 31 (4.4%) alkaloids, 27 (3.83%) terpenoids, 21 (2.98%) lignans and coumarins, and 11 (1.56%) tannins (Figure S1A). Additionally, GC–MS/MS analysis of VOCs identified 552 compounds (Table S2), of which 134 (24.28%) terpenoids and 108 (19.57%) esters were the most abundant VOCs, and 69 (12.5%) heterocyclic compounds, 58 (10.51%) alcohols, 42 (7.61%) hydrocarbons, 42 (7.61%) ketones, 22 (3.99%) aromatics, 29 (5.25%) aldehydes, 12 (2.17%) acids, and 11 (1.99%) amines were also characterized (Figure S1B). Principal component analysis (PCA) of the UPLC–MS/MS and GC–MS/MS datasets showed clear separation of the red-fleshed cultivar ‘RF’ in the score plots (Fig. 1B, C). To detect the metabolite accumulation patterns among the three cultivars, hierarchical cluster analysis was performed on the basis of the orthonormal peak area for each metabolite. A high degree of consistency of metabolite accumulation patterns was observed among the three biological replicates (Fig. 1D, E). Notably, most of the VOCs characterized in this work, including terpenoids, acids, and aldehydes, were downregulated in the ‘YF’ cultivar, which has white flesh (Fig. 1E).

### Differentially accumulated metabolite analyses

**Fig. 2:**
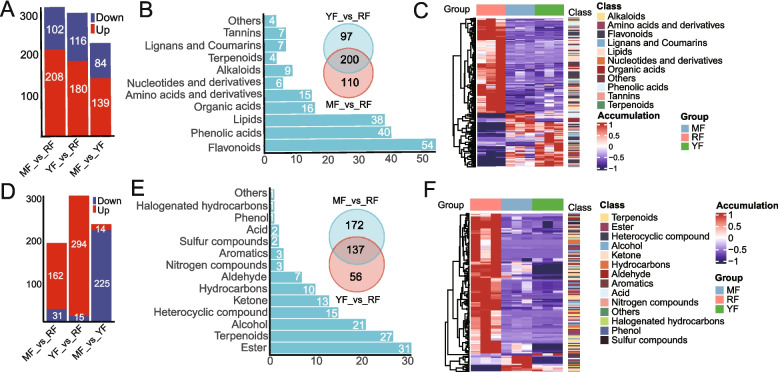
Identification of differentially abundant metabolites. **A** Comparison of differentially abundant metabolites among different groups. Here, three comparable groups were constructed, namely, MF_vs_RF, YF_vs_RF, and MF_vs_YF, and the annotated term “Down” refers to these metabolites that were downregulated in the ‘RF’ cultivar, and ‘YF’ and “Up” refer to those metabolites that were upregulated in the ‘RF’ and ‘YF’ cultivars; similarly, hereinafter, (**B**) identification of differentially abundant metabolites in the red-fleshed “RF” cultivar. Here, the common differentially abundant metabolites identified between the MF_vs_RF and YF_vs_RF groups are presented in a Venn diagram, and the classification of differentially abundant metabolites in the ‘RF’ cultivar is presented in a histogram. Similarly, (**C**) The classification and accumulation patterns of differentially abundant metabolites in the “RF” cultivar. **D** Comparison of VOCs among different groups. **E**, **F** Identification of volatile organic compounds (VOCs) (**E**) and the accumulation patterns of volatile organic compounds (VOCs) in the “RF” cultivar (**F**)

Differentially accumulated metabolites (DAMs) were identified via a fold-change threshold of ≥ 2 (upregulation) or ≤ 0.5 (downregulation), combined with VIP values (VIP ≥ 1) from the OPLS-DA (orthogonal projection to latent structures-discriminant analysis) model. Comparative analysis indicated substantial metabolic upregulation in the red-fleshed cultivar ‘RF’ compared to the two white-fleshed cultivars (‘MF’ and ‘YF’). Specifically, 208 (67%) and 180 (61%) metabolites exhibited increased accumulation in ‘RF’ in the MF_vs_RF and YF_vs_RF comparison groups, respectively (Fig. 2A). Notably, 139 metabolites (62%) were upregulated in the green-peeled, white-fleshed ‘YF’ cultivar relative to the red-peeled, white-fleshed ‘MF’ (Fig. 2A). In this study, we selected the ‘RF’ cultivar as our primary focus because of its characteristically high flavonoid content, a well-documented metabolic feature of red-fleshed apple varieties [15]. A total of 200 DAMs were commonly identified in ‘RF’, including 54 (27%) flavonoids, 40 (20%) phenolic acids, and 38 (19) lipids (Fig. 2B, Table S3). Most commonly identified DAMs, including lipids, flavonoids, and terpenoids, were significantly upregulated in the ‘RF’ cultivar (Fig. 2C). Interestingly, tannins were notably downregulated in the ‘RF’ cultivar (Fig. 2C).

For volatile organic compounds (VOCs), targeted profiling via GC–MS identified 193 and 309 differentially accumulated VOCs in ‘RF’ compared to ‘MF’ and ‘YF’, respectively (Fig. 2D). Notably, the majority of these VOCs (84% and 95%) were upregulated in the red-fleshed cultivar (Fig. 2D). In the MF_vs_YF comparison, 239 differentially accumulated VOCs were detected, with 225 compounds (94%) showing decreased accumulation in the green-peel, white-fleshed ‘YF’ cultivar (Fig. 2D). Additionally, 137 VOCs were consistently differentially accumulated in ‘RF’, particularly those enriched with ester, terpenoid and alcohol metabolites (Fig. 2E, Table S3). We also found that most VOCs, such as esters, terpenoids, and alcohols, were significantly increased in the ‘RF’ cultivar (Fig. 2F). Overall, integrated metabolomic analysis demonstrated that the majority of both nonvolatile and volatile metabolites were upregulated in red-fleshed apples.

### Characteristics of flavonoid accumulation in red-fleshed apple

**Fig. 3:**
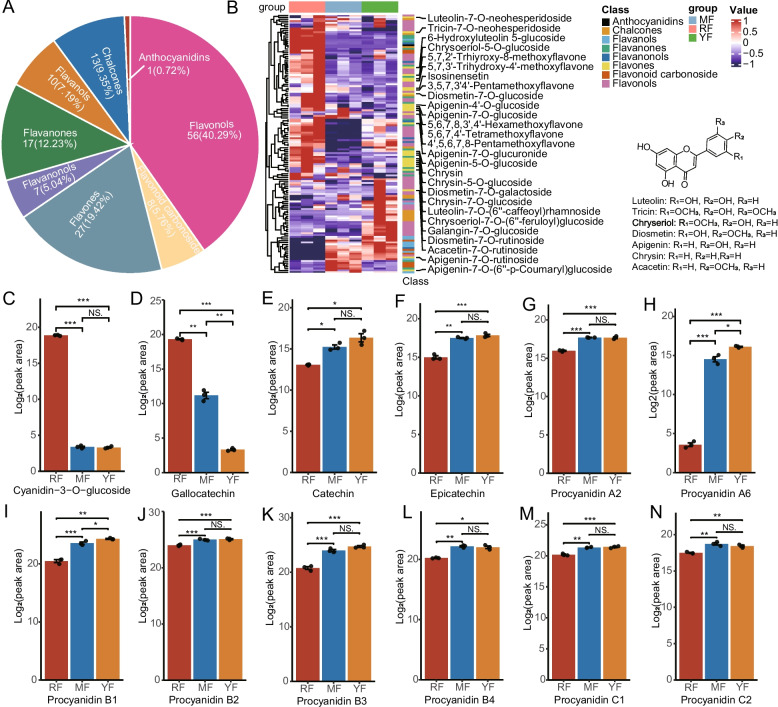
Flavonoid accumulation patterns in three apple cultivars. **A** Statistics of flavonoids identified in three apple cultivars on the basis of LC‒MS/MS data; (**B**) The accumulation pattern of flavonoids. Representative metabolites are marked on the side of the heatmap. The structural skeletons of flavones are presented, and R_1_, R_2_, and R_3_ refer to different chemical groups. **C**-**N** The accumulation patterns of anthocyanin (**C**), gallocatechin (**D**), catechin (**E**), epicatechin (**F**) and proanthocyanidins (**F**-**N**). The relative abundance of the metabolites was evaluated on the basis of the logarithm of the peak area of the LC‒MS/MS data. Differences were compared via a *t* test (“*”: *p* < 0.05; “**”: *p* < 0.01; “***”: *p* < 0.001; NS.: no significant difference, *n* = 3)

Red-fleshed apples have received increasing interest because of their high flavonoid content [42]. In this study, we conducted a detailed investigation of flavonoid accumulation patterns across three cultivars. Metabolomic profiling identified eight classes of flavonoids, with flavonols being the most abundant (56 compounds, 40.29%), followed by flavones (27, 9.42%), flavanones (17, 12.23%), chalcones (13, 9.35%), flavanols (10, 7.19%), flavanonols (7, 5.04%), flavonoid carbonosides (8, 5.76%), and anthocyanidins (1, 0.72%) (Fig. 3A). We found that most flavones, such as tricin-7-O-neohesperidoside, 6-hydroxyluteolin 5-glucoside, chrysoeriol-5-O-glucoside, apigenin-4'-O-glucoside, and diosmetin-7-O-galactoside, were markedly upregulated in the ‘RF’ cultivar (Fig. 3B). Notably, several polymethoxyflavones (PMFs), a class of specialized flavonoids known for their notable anticancer properties in plants such as apple and citrus, showed significantly increased in accumulation in red-fleshed cultivars. These included 3,5,7,3'4'-pentamethoxyflavone, 5,6,7,8,3',4'-hexamethoxyflavone, 4',5,6,7,8-pentamethoxyflavone, isosinensetin, and 5,6,7,4'-tetramethoxyflavone (Fig. 3B, Figure S2A). Only one anthocyanidin, cyanidin-3-O-glucoside, was identified and uniquely accumulated in the red-fleshed cultivar (Fig. 3A, C). In contrast, most flavanols, including catechin, epicatechin, epicatechin-epiafzelechin, catechin − catechin − catechin, 8,8' − methylenebiscatechin, and catechin-(7,8-bc)−4β-(3,4-dihydroxyphenyl)-dihydro-2-(3H)-one, were downregulated in the ‘RF’ cultivar (Fig. 3B, E, F and Figure S2B), with the exception of gallocatechin which was upregulated (Fig. 3D). Furthermore, the accumulation of procyanidins (A-, B-, and C-type) was substantially lower in ‘RF’ than in ‘MF’ and ‘YF’ (Fig. 3G-N). In summary, our findings revealed pronounced upregulation of flavonoids in red-fleshed apples, and the accumulation of poly-methylated flavone derivatives was particularly increased. Conversely, flavanols and proanthocyanidins presented significantly reduced accumulation in red-fleshed apples.

### Identification and functional annotation of DEGs through RNA-seq

**Fig. 4:**
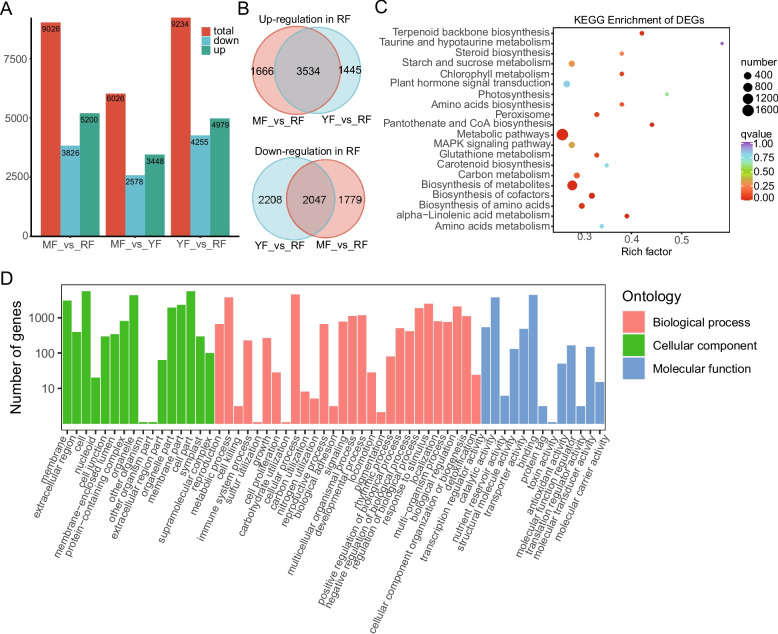
Transcriptomic analysis of three apple cultivars. **A** Number of differentially expressed genes (DEGs) in apple cultivars; (**B**) Venn diagram of DEGs commonly expressed in the ‘RF’ cultivar. The upregulated genes and downregulated genes are presented in a Venn diagram. **C** KEGG enrichment of the DEGs commonly expressed in the ‘RF’ cultivar. **D** GO (Gene Ontology) annotation of the DEGs commonly expressed in the ‘RF’ cultivar. The GO terms biological process (BP), cellular component (CC), and molecular function (MF) were annotated

To identify the genes responsible for the unique metabolite accumulation in red-fleshed apples, we conducted RNA sequencing on flesh tissues from three cultivars. Total RNA of the flesh was extracted from three cultivars, and nine high-quality cDNA libraries were further amplified and constructed. RNA-seq produced 45.1–52.2 million raw reads (average of 6.6 Gb of clean data per sample), with a Q30 percentage of 91.1–92.2% and an average GC content percentage of 47.4% (Table S5). All the reads were successfully mapped to the reference genome, with percentages of 87.83%−91.36%, and 84.19%−88.43% of the reads were uniquely mapped (Table S5). These results suggested that high-quality sequencing data can be used for further analyses. A total of 35,977 transcripts were identified and annotated using BLAST against the NCBI database, including 2,972 novel transcripts (Table S6). Differential expression analysis was performed based on fold change in FPKM and FDR values.. In the MF_vs_RF comparison, 9,026 DEGs were detected, with 5,200 upregulated and 3,826 downregulated in ‘RF’. The YF_vs_RF comparison revealed 9234 DEGs, including 4,979 upregulated and 4,255 downregulated in the ‘RF’. In the MF_vs_YF group, 6,026 DEGs were found, of which 3,448 were upregulated and 2,578 were downregulated in the ‘YF’ cultivar (Fig. 4A). Further analysis identified 3,534 upregulated and 2,047 downregulated transcripts consistently associated with the red-fleshed cultivar (Fig. 4B). All DEGs in the ‘RF’ cultivar were further subjected to GO and KEGG analyses. KEGG pathway analysis revealed significant enrichment of metabolic and biosynthetic pathways, such as general metabolic pathways, terpenoid backbone biosynthesis, starch and sucrose metabolism, and biosynthesis of metabolites, among the differentially expressed transcripts. (Fig. 4C). GO is also used for DEG functional classification, including biological process (BP), molecular function (MF), and cellular component (CC) categories. In BP, 28 GO terms were maximally enriched, including metabolic process, cellular process, and cellular component organization and biogenesis (Fig. 4D). In MF, catalytic activity and binding and transcription regulator activity were the most common GO terms (Fig. 4D). In CC, the GO terms “cell part”, “protein complex”, and “organelle part” were significantly enriched (Fig. 4D).

### Expression of genes involved in flavonoid biosynthesis

**Fig. 5:**
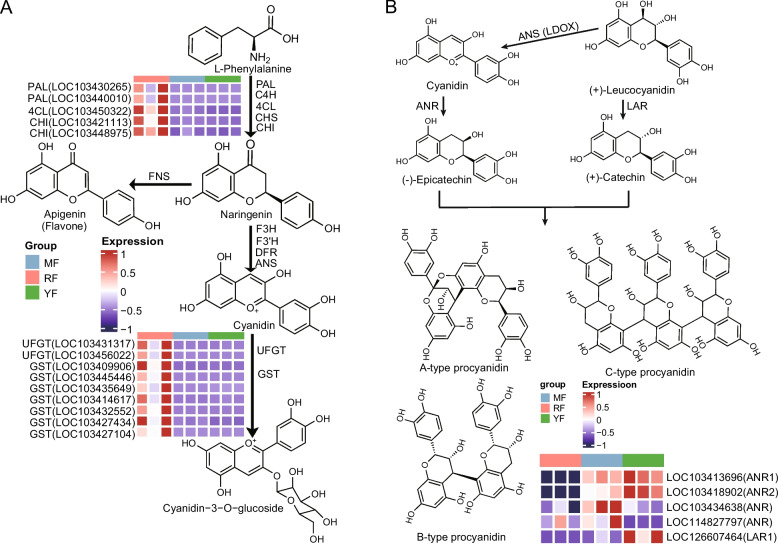
Identification of DEGs involved in anthocyanin and PA biosynthesis. **A** Identification of the DEGs involved in the synthesis of flavones and anthocyanins in apple. The expression patterns of the key DEGs are presented via a heatmap. PAL: L-phenylalanine ammonia lyase; 4CL: 4-coumarate-CoA ligase; CHI: chalcone isomerase; FNS: flavone synthase; F3H: flavonoid 3-hydroxylase; F3'H: flavonoid 3'-hydroxylase; DFR: dihydroflavonol 4-reductase; ANS: anthocyanidin synthase; UFGT: UDP glucose: flavonoid-3-O-glucosyltransferase; GST: glutathione transferase. **B** Key genes involved in the synthesis of PAs. LAR (leucoanthocyanidin reductase) and ANR (anthocyanidin reductase) are key genes responsible for the synthesis of PAs, and their expression patterns are presented in a heatmap

In this work, we observed a marked enrichment of flavones in the red-fleshed cultivar ‘RF’ (Fig. 3A). Therefore, we further investigated the expression patterns of such genes related to the flavonoid synthesis pathway. As a result, the expression levels of *PAL*, *4CL*, and *CHI* responsible for the synthesis of flavanone (naringenin), a substrate for further hydrogenation reactions through the catalytic activity of *FNS*, were significantly increased in the ‘RF’ cultivar (Fig. 5A). This supports the increased accumulation of flavones, which subsequently undergo further modifications such as hydroxylation, methylation, and glycosylation. Although cyanidin − 3 − O − glucoside, the only anthocyanin identified in this study, was exclusively enriched in ‘RF’, the expression levels of *F3H*, *F3'H*, *DFR*, and *ANS*, such genes typically involved in cyanidin biosynthesis, were not significantly upregulated (Fig. 5A). Interestingly, the remarkably high expression of two *UFGT* genes responsible for the glycosylation of cyanidin and seven *GST* genes responsible for anthocyanin vacuole transport contributed to anthocyanin accumulation in the ‘RF’ cultivar (Fig. 5A). Another notable finding was the significant downregulation of proanthocyanidins (PAs) accumulation in the red-fleshed cultivar (Fig. 3G-N). We further investigated the expression patterns of *ANR* and *LAR*, which are the key genes involved in the synthesis of proanthocyanidins in apple [26]. The results indicated that two *ANR* genes (LOC103413696 and LOC103418902) and an *LAR1* gene (LOC126607464) were much more highly expressed in the white-fleshed cultivars ‘MF’ and ‘YF’ (Fig. 5B). Collectively, our findings suggest three distinct regulatory mechanisms in red-fleshed apples: (1) enhanced activity of early flavonoid pathway enzymes (PAL, CHS, CHI, etc.) promotes flavone and derivative accumulation; (2) glycosyltransferases (*e.g*., UFGT) and vacuolar transporters (*e.g*., GSTs) mediate anthocyanin accumulation; and (3) suppressed expression of the *LAR* gene and *ANR* gene potentially limits proanthocyanidin biosynthesis.

### Identification of differentially expressed transcription factors (TFs)

**Fig. 6:**
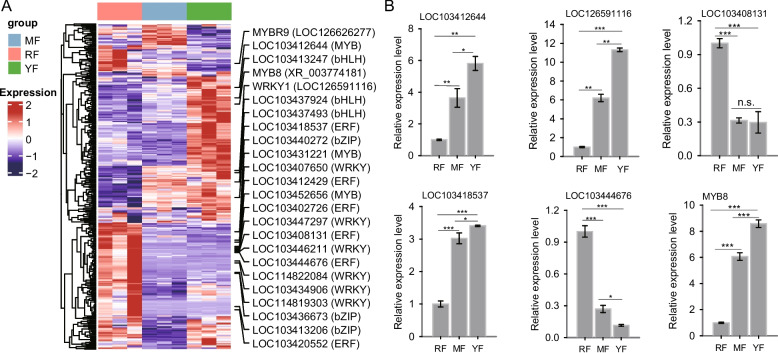
Identification of differentially expressed TFs among the three apple cultivars. **A** The expression patterns of the TFs identified on the basis of the DEGs are presented in a heatmap. Some members of the MYB family, bHLH family, bZIP family, WRKY family, and ERF family are labeled on the side of the heatmap. **B** The transcriptomic expression of TFs was verified via RT‒qPCR. Six TFs, including MYB family members (MYB8 and LOC103412644), ERF family members (LOC103408131, LOC103418537, LOC103444676), and WRKY family members (LOC126591116), were confirmed via RT‒qPCR. Differences are compared via a *t* test (“*”: *p* < 0.05; “**”: *p* < 0.01; “***”: *p* < 0.001; n.s.: no significant difference)

It was speculated that cyanidin − 3 − O − glucoside contributed to red-fleshed coloration due to its greater accumulation in the ‘RF’ cultivar (Fig. 3C). TFs involved in apple anthocyanin biosynthesis regulation, such as those in the WRKY family, MYB family, bZIP family and NAC family, have been well investigated [15, 20, 33]. In this study, we focused on the TFs involved in the regulation of anthocyanin biosynthesis. Based on the differentially expressed genes (DEGs) identified in the ‘RF’ cultivar, key TFs from the bZIP, WRKY, MYB, bHLH, and ERF families were selected for further analysis. The results s that multiple TFs were oppositely differentially expressed between the ‘RF’ and ‘YF’ cultivars (Fig. 6A). We further verified the expression levels of the TFs via RT‒qPCR on the basis of selected TF candidates, for example, the transcription factor MYB family genes LOC103412644, MYB8, and WRKY1, which are upregulated in the ‘YF’ cultivar; the ERF family gene LOC103418537, which is significantly downregulated in the ‘RF’ cultivar; and the two ERF genes LOC103408131 and LOC103444676, which are upregulated in the ‘RF’ cultivar (Fig. 6B). Collectively, the RT‒qPCR validation confirmed the reliability of our transcriptomic data, and the identified differentially expressed TFs are likely to play important roles in regulating anthocyanin accumulation in red-fleshed apples.

### MdERF072 is responsible for anthocyanin accumulation

**Fig. 7:**
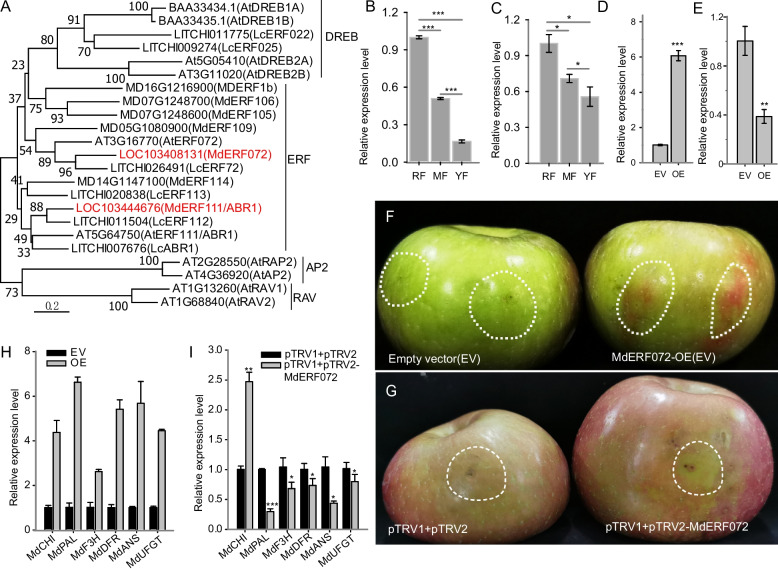
Functional verification of *MdERF072* in apple. **A** Phylogenetic analysis of *MdERF072* and *MdERF111*/*ABR1* and related AP2/ERF TFs from different species. Here, *MdERF072* and *MdERF111*/*ABR1* were cloned from the ‘RF’ cultivar, and the putative protein sequences were used to perform multiple sequence alignment and to construct a maximum likelihood based on the Dayhoff model. Different subfamilies of AP2/ERF TFs were obtained from *Arabidopsis* (At), apple (Md) and litchi (Lc). The accession numbers of the AP2/ERF TFs were retrieved from such a database (http://plants.ensembl.org/index.html; http://www.sapindaceae.com/index.html). **B**, **C** Expression patterns of *MdERF072* (**B**) and *MdERF111*/*ABR1* (**C**) in the pericarp of the ‘RF’, ‘MF’, and ‘YF’ cultivars. **D** The expression level of *MdERF072* in ‘Fuji’ apple that was used for the transient injection experiment compared with the empty vector control (EV). **E** The expression level of *MdERF072* in apple that was performed by virus induced gene silencing (VIGS) assay. pTRV1 and pTRV2 were used as control. **F**, **G** Phenotypes of infiltrated apple fruits with EV and MdERF072 overexpression (**F**) and VIGS assay (**G**). **F**, **I** Transcript levels of genes related to anthocyanin biosynthesis in infiltrated apple fruits from MdERF072 overexpression (OE) group and VIGS group. Differences were compared via a *t* test (“*”: *p* < 0.05; “**”: *p* < 0.01; “***”: *p* < 0.001)

In this work, we focused on ERF family TFs because previous studies revealed that *MdERF109*, *MdERF1B*, and *MdERF38* regulate anthocyanin biosynthesis in apple [5, 21, 22]. We cloned two ERF family genes, LOC103408131 (ID in *Malus domestics* v1.1: MD11G1306500) and LOC103444676 (MD10G1032000), both of which were significantly upregulated in the ‘RF’ cultivar. Phylogenetic analysis indicated that these two *ERF* genes belong to the ERF subfamily, of which LOC103444676 is close to *AtERF111*/*ABR1* in *Arabidopsis* (named *MdERF111*/*ABR1* in this work) and LOC103408131 is close to *AtERF072* in *Arabidopsis* (named *MdERF072* in this work) (Fig. 7A). Furthermore, the expression patterns of *MdERF111*/*ABR1* and *MdERF072* were analyzed in the peels of three apple cultivars, and both genes were upregulated in the ‘RF’ and ‘MF’ cultivars (Fig. 7B, C). However, only *MdERF072* presented the same expression pattern between the ‘MF’ and ‘YF’ cultivars, both of which are white-fleshed apples (Fig. 6B, LOC103408131). We therefore hypothesized that *MdERF072* may act as a positive regulator of anthocyanin accumulation specifically in red-fleshed cultivars. *MdERF072* was subsequently cloned and inserted into a binary vector and transiently expressed in ‘Fuji’ apple (MdERF072-OE) (Fig. 7D). The results revealed that, compared with the empty vector (EV), anthocyanin significantly accumulated around the injection orifice (Fig. 7F). The expression levels of genes associated with the anthocyanin biosynthesis pathway were detected via RT‒qPCR, which revealed that most genes, such as *MdPAL*, *MdCHI*, *MdF3H*, *MdDFR*, *MdANS*, and *MdUFGT,* were significantly upregulated in MdERF072-OE apple (Fig. 7H). Silencing *MdERF131* expression through VIGS experiments suppressed anthocyanin biosynthesis and downregulated the expression of other related genes, with the exception of MdCHI (Fig. 7E, F, I). These results preliminarily revealed that *MdERF072* increased anthocyanin accumulation in apple.

## Discussion

The apple tree is one of the most widely cultivated and economically important temperate fruit crops, serving as a key source of flavonoids and other nutrients in the human diet [43]. While apple peels contain nearly half of all flavonoids and contain nearly all flavonols and anthocyanins in whole apples. Given that consumers typically consume significantly more flesh than peel, red-fleshed apple cultivars represent a valuable means to increase dietary flavonoid consumption for health promotion [14]. In recent years, interest in developing a commercial red-fleshed apple has increased since a red-fleshed apple (*Malus sieversii*) was discovered by the botanist Niedzwetzky [44]. In this study, we applied a comprehensive metabolomics approach to compare volatile and nonvolatile metabolites among three commercially relevant apple cultivars (‘Red Love 119–07’, ‘YanFu 8’, and ‘Venus Golden’) which exhibit distinct peel and flesh coloration, thereby uncovering metabolic profiles unique to red-fleshed apples. A total of 705 volatile and 552 nonvolatile metabolites were identified and characterized in this work (Fig. 1D, E). Previous studies on apple metabolites have typically relied on targeted methods such as UPLC‒tandem mass spectrometry (UPLC‒tandem mass spectrometry (UPLC‒MS/MS) and SPME‒GC‒MS (SPME‒MS/MS) methodologies [37, 45], focusing on limited classes of compounds like phenolic acids, anthocyanins, dihydrochalcones, organic acids, and specific flavonoids [46]. In contrast, our study offers a more comprehensive comparison of the differences in metabolite accumulation among three common apple varieties. Our results revealed that the flavonoid content was significantly greater in the red-fleshed apple cultivar ‘Red Love 119–07’ than in ‘YanFu8’ and ‘Venus Golden’, with notable upregulation and accumulation of PMFs in red-fleshed apple plants (Fig. 3B, Figure S2A). PMFs are a subclass of flavones featuring multiple methoxy (-OCH₃) groups on the benzopyrone backbone and are recognized as bioactive natural products with demonstrated anticancer properties [47]. The predominant PMFs include tangeretin, nobiletin, and 5-demethylnobiletin, and these bioactive compounds are widely distributed in citrus (*Citrus spp*.), strawberry, and berry cultivars (e.g., grape and blueberry) [48–50]. In this study, we identified five PMFs that were significantly accumulated in red-fleshed 'Red Love 119–07' apples (Figure S2A). Transcriptome analysis revealed elevated expression levels of key upstream genes involved in PMF biosynthesis, including *PAL*, *C4H*, *4CL*, *CHS*, and *CHI* (Fig. 5A). This coordinated upregulation of these genes likely contributes to the observed PMF accumulation phenotype in red-fleshed ‘Red Love 119–07’ [51].

Notably, our analysis revealed significant upregulation of the majority of VOCs in the red-fleshed apple cultivar 'Red Love 119–07', with particularly pronounced accumulation observed in the ester, terpenoid, and alcohol classes (Fig. 2D, E, F). The aromatic profile of apples is characterized by a complex mixture of VOCs, predominantly esters, alcohols, aldehydes, terpenoids, and other secondary metabolites [35]. Previous studies have shown that aldehydes (*e*.*g*., hexanal and 2-hexenal) accumulate at high level in immature apple fruit, while the VOC profile shifts from aldehyde-dominated to ester- and alcohol-rich during fruit ripening [52, 53]. Consistent with this, our data indicate that esters, terpenoids, and alcohols collectively account for more than 50% of the total VOCs in ripe apple fruit, whereas aldehydes represent merely 5% of the volatile profile (Figure S1B). In apples, alcohols and esters constitute the primary flavor determinants; however, the distinct aromatic profiles of different cultivars arise from variations in the composition and concentration of these compounds, for example, key ester components include ethyl 2-methylbutanoate, 2-methylbutyl acetate, and hexyl acetate, whereas major alcohols include 2-methyl-1-butanol, 1-hexanol, and (*Z*)−3-hexenol [35]. In our work, we also found that esters such as ethyl 2-methylbutanoate, 2-methylbutyl acetate, hexyl 2-methylbutanoate, and ethyl 3-methylbutanoate were markedly enriched in the red-fleshed ‘Red Love 119–07’ cultivar (Table S4). These findings suggest that these esters may play a key role in defining the unique flavor profile of red-fleshed apples.

Another distinctive feature observed in our results was the significantly lower proanthocyanidin content in the red-fleshed apple 'Red Love 119–07' compared to the white-fleshed cultivars 'YanFu 8' and 'Venus Golden' (Fig. 3G-N). This reduction can be partly attributed to the decreased accumulation of flavan-3-*ols*, which serve as the monomeric units of proanthocyanidins (Fig. 3E, F and Figure S2B). Transcriptomic data revealed that the *LAR1* and *ANR* genes, key genes responsible for the biosynthesis of catechin and epicatechin, are downregulated in ‘Red Love 119–07’ apples (Fig. 5B), providing a plausible molecular explanation for the low proanthocyanidin accumulation in red-fleshed apples [26]. Surprisingly, however, the accumulation of gallocatechins (GCs) was significantly increased (Fig. 3D). In plants, the biosynthesis of GCs is dependent on 3' and 5' hydroxylation reactions of the B-ring catalyzed by the flavonoid 3',5'-hydroxylase (F3′5'H), which belongs to the cytochrome P450 family [54]. However, some studies have suggested that functional F3′5'H enzymes are absent in apples [55]. Therefore, alternative hydroxylases in apples may catalyze the 3' and 5' hydroxylation of dihydrokaempferol, and the high expression of these genes in red-fleshed cultivars could drive the increased accumulation of GCs [56].

The most distinctive feature of red-fleshed apples is their high anthocyanin content, which confers vivid red pigmentation to both peel and flesh tissues. Current studies have identified two distinct types that control anthocyanin accumulation in apple flesh, each of which is regulated by separate MYB transcription factors. In type I red-fleshed apples derived from Niedzwetzky’s apple, MdMYB10 regulates the accumulation of anthocyanins throughout the plant (flesh, skin, flowers, leaves, and stems). In contrast, type II apples (‘Surprise’ progeny) develop red pigmentation only in the flesh, which is controlled by MYB110a [44]. MYB10 is expressed early during fruit maturation, whereas MYB110a is expressed in later stages [57]. In addition to the well-characterized MYB family genes, emerging evidence indicates that AP2/ERF family transcription factors contribute to flesh-specific anthocyanin biosynthesis in apple [21, 22]. In this work, two AP2/ERF family members (*MdERF111*/*ABR1* and *MdERF072*) belonging to the ERF subfamily were upregulated in the red-fleshed ‘RF’ cultivar (Figs. 6B and 7A). It is worth noting that orthologs of *MdERF111*/*ABR1* in other plant species are known to function as hubs in ABA and Ca^2^⁺ signaling pathways and in abiotic stress responses [58, 59]. The biological function of *MdERF072* in promoting anthocyanin accumulation in apples was validated through transient expression and VIGS assays, which is probably able to regulate the expression of key anthocyanin biosynthetic enzyme-encoding genes (Fig. 7E, F). Several studies have revealed that AP2/ERF family genes may increase (or suppress) the regulatory activity of anthocyanin biosynthesis activators such as MYB TFs, thereby modulating anthocyanin production [21–23]. In the next work, we aim to determine how *MdERF072* regulates the biosynthesis of anthocyanin in apples.

## Conclusions

Integrated metabolomic and transcriptomic analyses revealed unique metabolic features of red-fleshed apples. Red-fleshed apples showed widespread upregulation of both non-volatile metabolites and volatile organic compounds. Notably, flavonoid metabolism was specifically reprogrammed: anthocyanins and PMFs accumulated markedly, whereas flavanol and proanthocyanidin biosynthesis was suppressed in red-fleshed apple. Transcriptomic data indicated that upregulation of anthocyanin modification and transport genes (e.g., *UFGT*, *GSTs*) promoted anthocyanin accumulation, while downregulation of proanthocyanidin synthesis genes (*ANR*, *LAR*) limited their production. Furthermore, the ERF-family transcription factor *MdERF072* was specifically highly expressed in red-fleshed varieties and functionally validated as an important activator of anthocyanin pathway genes. These results offer a theoretical foundation and genetic resources for improving apple fruit color.

## Supplementary Information

Supplementary Material 1.

Supplementary Material 2.

## Data Availability

The data presented in this study were deposited into the NCBI, and the accession ID is PRJNA979000.
