# Supplementary figures and images for: Metabolic profiling of red-fleshed apple and functional characterization of MdERF072 in anthocyanin biosynthesis
Source: BMC Plant Biol. 2025 Dec 23;26:158. doi: 10.1186/s12870-025-07987-5 (PMC12837208; doi:10.1186/s12870-025-07987-5)

A

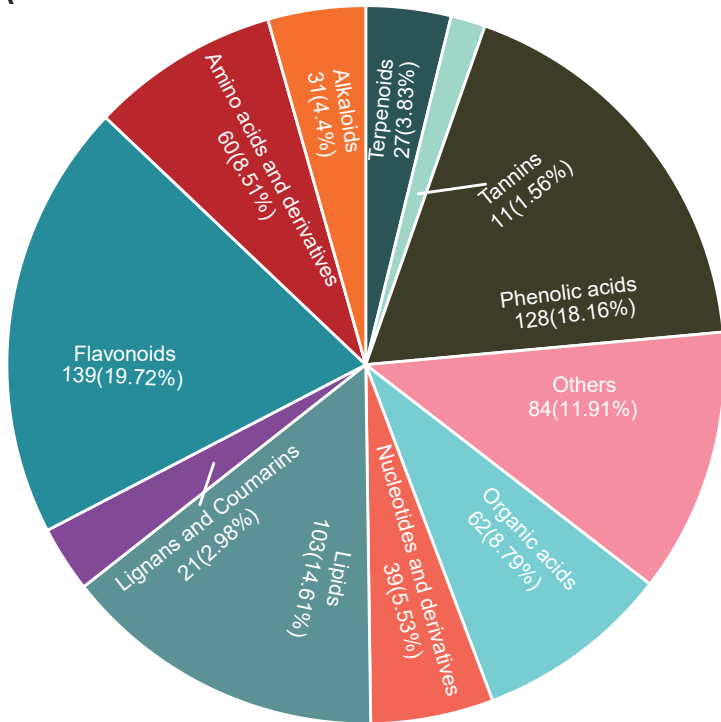

B

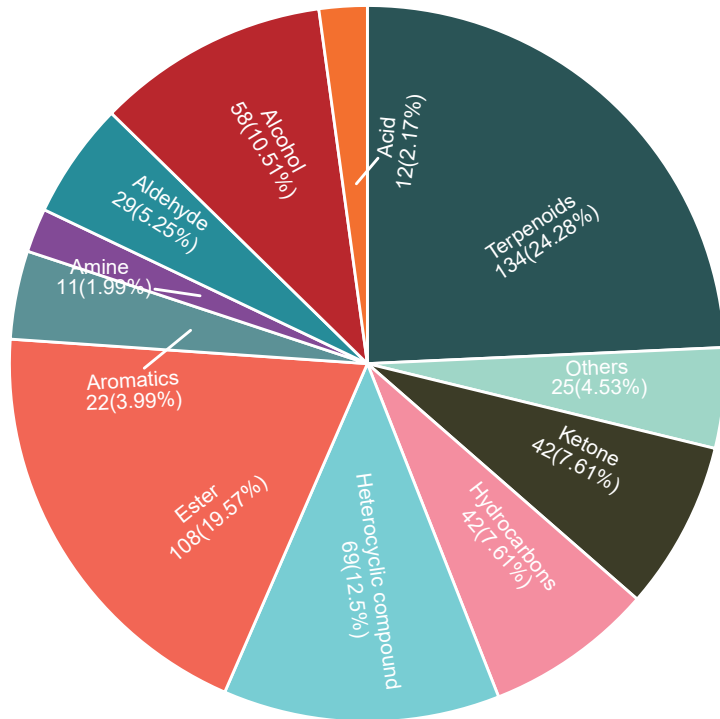

Supplement: Supplementary file 1 — Supplementary Material 1. [file 12870_2025_7987_MOESM1_ESM.zip › Figure S1.pdf]

A

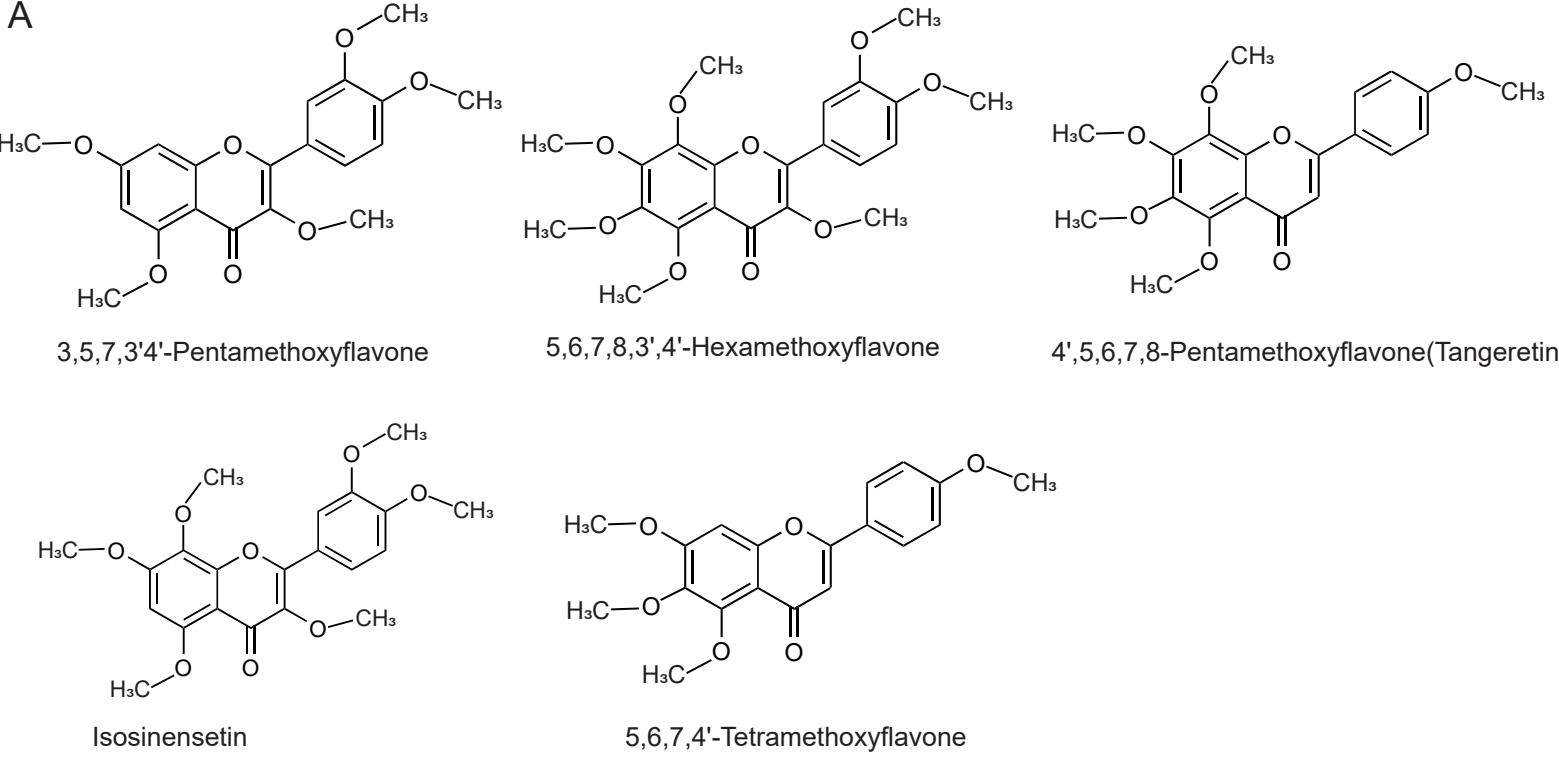

B

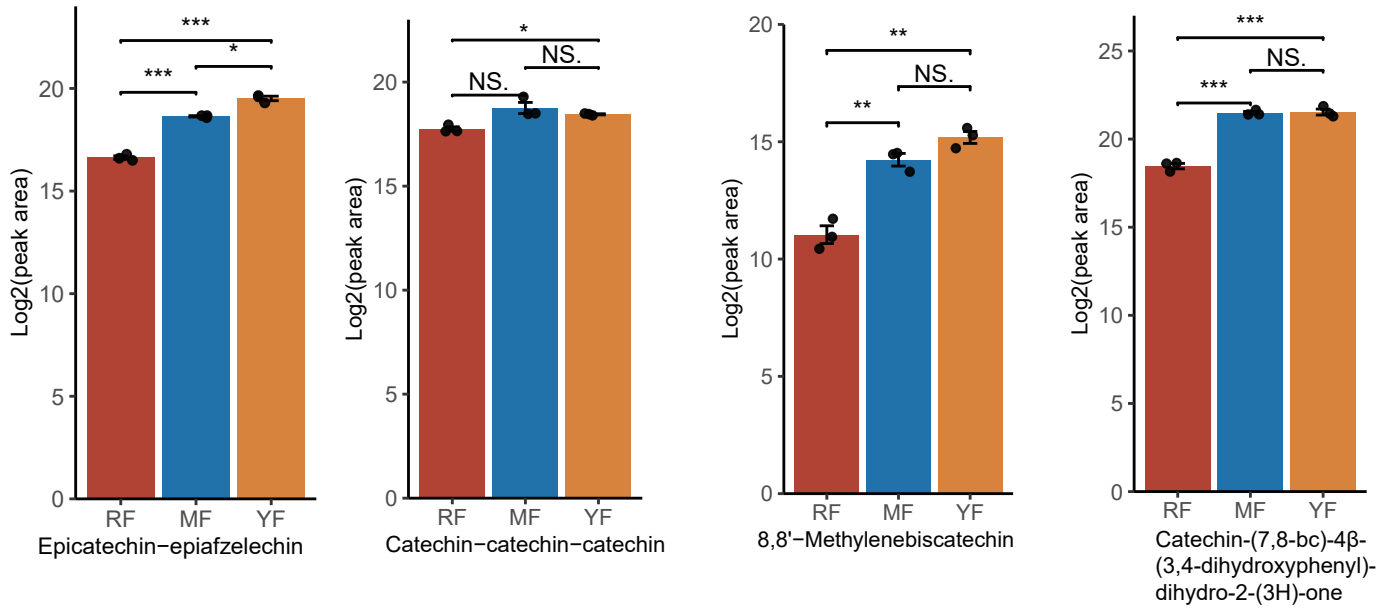

Supplement: Supplementary file 1 — Supplementary Material 1. [file 12870_2025_7987_MOESM1_ESM.zip › Figure S2.pdf]
